# Supplementary figures and images for: Stevioside Enhances the Anti-Adipogenic Effect and β-Oxidation by Activating AMPK in 3T3-L1 Cells and Epididymal Adipose Tissues of db/db Mice
Source: Cells. 2022 Mar 23;11(7):1076. doi: 10.3390/cells11071076 (PMC8997985; doi:10.3390/cells11071076)

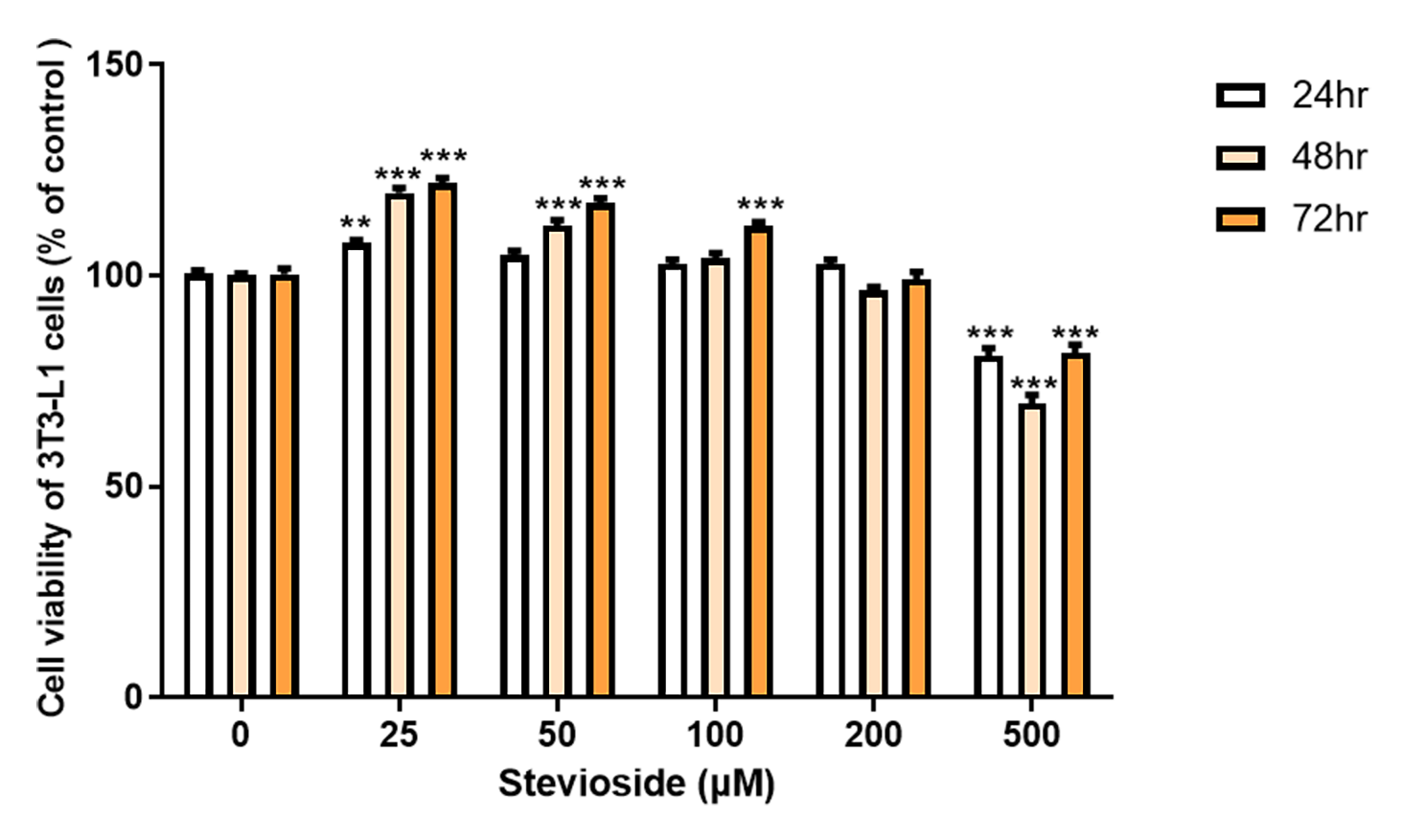

Supplement: Supplementary file 1 [file cells-11-01076-s001.zip › cells-1643772-supplementary.tif]
